# Editorial: Neutrophil-Mediated Skin Diseases: Immunology and Genetics

**DOI:** 10.3389/fimmu.2019.02377

**Published:** 2019-10-09

**Authors:** Angelo V. Marzano, Dan Lipsker, Massimo Cugno

**Affiliations:** ^1^Dermatology Unit, Fondazione IRCCS Cà Granda Ospedale Maggiore Policlinico, Milan, Italy; ^2^Department of Physiopathology and Transplantation, Università Degli Studi di Milano, Milan, Italy; ^3^Faculté de Médecine, Université de Strasbourg et Clinique Dermatologique, Hôpitaux Universitaires, Strasbourg, France; ^4^Internal Medicine Unit, Fondazione IRCCS Cà Granda Ospedale Maggiore Policlinico, Milan, Italy

**Keywords:** neutrophil, skin—immunology, innate immunity, genetics, neutrophilic dermatoses (NDs), autoinflammation

Neutrophils are involved in the effector phase of the host defense against micro-organisms and have a major role in the innate immune response but they also act in the modulation of the adaptive immunity as well as in orchestrating the response of the immune system to other triggers such as severe injury and trauma (Mortaz et al.). The deregulation of neutrophil function and their hyperactivity can lead to inflammation and tissue damage as seen in neutrophilic dermatoses that are a group of diseases due to accumulation of neutrophils in the skin and less frequently in internal organs (Marzano et al.). The systemic involvement, which may be sometimes severe, has led to coining the term “neutrophilic diseases.” The prototype of neutrophilic diseases are pyoderma gangrenosum and Sweet's syndrome (Marzano et al.; Heath and Ortega-Loayza) but some authors suggest to include hidradenitis suppurativa as well (Tricarico et al.; Vossen et al.; Frew), though there is no full agreement on this point; for all these entities an important autoinflammatory component has been demonstrated in their pathogenesis. Moreover, the spectrum of neutrophilic diseases is broad; it comprises truly systemic diseases such as Behçet's disease (Leccese and Alpsoy), but also psoriasis where neutrophils play an important role in the pathophysiology (Le et al.; Wannick et al.) or the inflammatory immunological response of leprosy (Schmitz et al.). The present issue is focused on the interplay between immunology and genetics in neutrophil-mediated diseases, highlighting the close links with the group of autoinflammatory diseases. The latter are characterized by recurrent episodes of sterile inflammation in the affected organs with neutrophils involved as leading cells, and are due to mutations in genes regulating the innate immunity. The recognition of several monogenic diseases which can present with neutrophilic skin diseases, such as CAPS (cryopyrin-associated periodic syndromes), DIRA (deficiency of IL-1 receptor antagonist), DITRA (deficiency of IL-36 receptor antagonist), and PAPA (pyogenic sterile arthritis, pyoderma gangrenosum, acne), has led to an improved understanding of the possible mechanisms of polygenic non-mendelian inherited neutrophilic skin diseases (Marzano et al.; Heath and Ortega-Loayza; Tricarico et al.; Vossen et al.). An increasing body of evidence supports the role of pro-inflammatory cytokines like interleukin (IL)-1-beta, IL-17, and tumor necrosis factor (TNF)-alpha in the pathophysiology of neutrophilic diseases similarly to classic monogenic autoinflammatory diseases, suggesting common physiopathological mechanisms. Moreover, mutations of several genes involved in autoinflammatory diseases are likely to play a role in the pathogenesis of sporadic neutrophilic diseases, giving rise to regarding them as a spectrum of polygenic autoinflammatory conditions (Marzano et al.; Heath and Ortega-Loayza; Tricarico et al.; Vossen et al.). Indeed, mutations of *PSTPIP1* (proline-serine-threonine phosphatase interacting protein 1), the gene involved in PAPA, as well as of a number of other genes involved in classic autoinflammatory diseases have been demonstrated in both isolated and syndromic forms of pyoderma gangrenosum, whose prototype is PASH (pyoderma gangrenosum, acne, suppurative hidradenitis), as well as in neutrophilic diseases in general. At present, classic regimens such as systemic glucocorticosteroids and immunosuppressants are the mainstay of treatment while biologic drugs are reserved for refractory cases. We can thus hope that the precise elucidation of the immunology and genetics of neutrophil-mediated diseases will pave the way to pathogenesis-driven treatments and the development of new drugs specifically targeting the inflammatory pathways involved in those entities.

## Author Contributions

All authors listed have made a substantial, direct and intellectual contribution to the work, and approved it for publication.

### Conflict of Interest

The authors declare that the research was conducted in the absence of any commercial or financial relationships that could be construed as a potential conflict of interest.

